# The Association Between the Use of Antenatal Care Smartphone Apps in Pregnant Women and Antenatal Depression: Cross-Sectional Study

**DOI:** 10.2196/11508

**Published:** 2018-11-29

**Authors:** Yushi Mo, Wenjie Gong, Joyce Wang, Xiaoqi Sheng, Dong R Xu

**Affiliations:** 1 XiangYa School of Public Health Central South University Changsha China; 2 Hunan Provincial Maternal and Child Health Hospital Changsha China; 3 Sun Yat-sen Global Health Institute School of Public Health and Institute of State Governance Sun Yat-sen University Guangzhou China

**Keywords:** antenatal care, antenatal depression, app, mobile phone

## Abstract

**Background:**

Antenatal care smartphone apps are increasingly used by pregnant women, but studies on their use and impact are scarce.

**Objective:**

This study investigates the use of antenatal care apps in pregnant women and explores the association between the use of these apps and antenatal depression.

**Methods:**

This study used a convenient sample of pregnant women recruited from Hunan Provincial Maternal and Child Health Hospital in November 2015. The participants were surveyed for their demographic characteristics, use of antenatal care apps, and antenatal depression. Factors that influenced antenatal pregnancy were analyzed using logistic regression.

**Results:**

Of the 1304 pregnant women, 71.31% (930/1304) used antenatal care apps. Higher usage of apps was associated with urban residency, nonmigrant status, first pregnancy, planned pregnancy, having no previous children, and opportunity to communicate with peer pregnant women. The cutoff score of the Edinburgh Postnatal Depression Scale was 10, and 46.11% (601/1304) of the pregnant women had depression. Logistic regression showed that depression was associated with the availability of disease-screening functions in the apps (odds ratio (OR) 1.78, 95% CI 1.03-3.06) and spending 30 minutes or more using the app (OR 2.05, 95% CI 1.19-3.52). Using apps with social media features was a protective factor for antenatal depression (OR 0.33, 95% CI 0.12-0.89).

**Conclusions:**

The prevalence of the use of prenatal care apps in pregnant women is high. The functions and time spent on these apps are associated with the incidence of antenatal depression.

## Introduction

With the advent of the information age, the use of smartphones and apps is becoming more common. It is predicted that the world’s smartphone ownership rate will reach 66% in 2018, and China will have 1.3 billion smartphone users [1]. Meanwhile, the number of global app downloads has exceeded to 175 billion in 2017, with China becoming the world’s largest smartphone app market. In the 4^th^ quarter of 2017 alone, the usage time of Chinese users of iOS, Google Play, and third-party Android apps reached 200 billion hours [2]. China’s huge smartphone user base and high accessibility of Wi-Fi or 4G have made people’s frequent use of mobile apps a reality, and the use of these apps in health care is worthy of attention. As of December 2016, the number of people using the Internet for health purposes in China reached 195 million, accounting for 26.6% of all Internet users. Among them, the medical information inquiry use rate was the highest, accounting for 10.8% [3]. Consultation in obstetrics and gynecology was also the most used service in a leading Web-based medical consultation app in China [4]. Due to high demand and adherence, pregnant women have become a key target group for app developers.

Antenatal care apps (acAPPs) are a type of smartphone apps that provide prenatal care services and information targeting prepregnant and pregnant women. It is a new way of channeling information and interpersonal interaction in the current era of mobile technology development, which has been widely accepted [5,6]. A study in the United States showed that apps for women’s health and pregnancy accounted for 7% of all kinds of apps [7]. We found 110 maternal health-related apps on the Chinese app market. Functions of these apps were diverse although the provision of maternity-related information to pregnant women was the major one. This form of health guidance is superior to traditional methods and can even improve the quality of pregnancy care in areas with scarce medical resources [5,8-10]. Many apps also have social functions, professional counseling functions, and special tools, such as calculation of fetal movement, calculation of due date, and measurement of changes in body weight. These self-monitored health status functions can be used to track pregnant women’s health [5,8,11,12].

Although acAPPs are widely used, research on the relationship of such use with user health and especially mental health is scarce. At present, existing studies mainly focus on the use of apps for monitoring or intervening mental disorders. Sensors and apps of smartphones have been used to predict daily mood [13,14], detect depression [15-18], intervene depression [19], relieve pressure [20], and treat depression [18,19,21]. Mental health and app-related researches are even rarer, and their use and effect evaluation among pregnant women have not been reported. Pregnancy is a period of high incidence of mental disorders, which increases not only the risk of postpartum depression but also its severity [22-25].

There may be intergenerational negative effects from both physical and environmental aspects. Therefore, gestational depression has received much attention in recent years and was included in the category of perinatal depression by the Diagnostic and Statistical Manual of Mental Disorders, Fifth Edition, in 2013. The use period of acAPPs overlaps with the occurrence of gestational depression. Whether there is a certain relationship between the two and what that relationship may be are the factors not only worthy of researchers’ attention in the field of maternal and child health but also important information for acAPP developers.

This study investigates the use of acAPPs in pregnant women at a specialized hospital for obstetrics and gynecology, examines antenatal depression, and explores the correlation between the use of acAPPs and mental health during pregnancy. These results can provide direction and theoretical basis for the development of future acAPPs.

## Methods

### Sample

Participants (N=1304) were pregnant women who visited the maternity department of the Maternal and Child Health Hospital of Hunan Province from November 16, 2015, to November 21, 2015.

### Procedure

**Figure 1:**
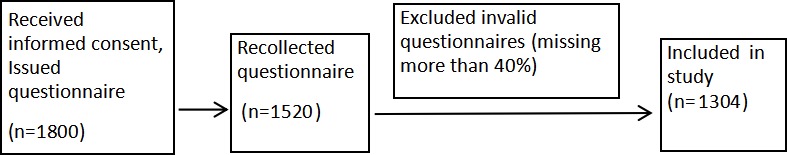
Flow of participants.

A convenience sampling approach was employed. During the investigation period, we explained the research purpose, content, and possible risks and benefits to all pregnant women who visited the Maternity Department of the Hunan Provincial Maternal and Child Health Hospital and invited them to participate in the survey. Questionnaires were issued to pregnant women aged 18 years and over who gave verbal consent. Women who were unable to understand the contents of the questionnaire were excluded from this study. We issued 1800 questionnaires, recollected 1520 questionnaires, and ultimately determined that 1304 questionnaires were valid, corresponding to an effective recovery rate of 72.44% (1304/1800). Flow of participants is presented in Figure 1. The study has been approved by the institutional review board of the Institute of Nursing and Behavioral Medicine Research, School of Nursing, Central South University (# 2015062).

### Measures

A self-developed questionnaire, optimized after preinvestigation, was used to collect information on demographics (eg, birth year, ethnicity, type of birthplace and residence, migrant population status, family income, and educational levels) and acAPP usage (eg, acAPP download channel, start usage time, duration of each use, frequency of use, and common functions).

The Edinburgh Postnatal Depression Scale (EPDS) is commonly used to screen for perinatal depression. It is a 10-item self-rated questionnaire, with each item scored from 0 to 3, giving a score ranging from 0 to 30. EPDS used to screen for antenatal depression in this study was translated by Wang Yuqiong [26], and several studies [27-30] have validated that it can be used for perinatal depression screening, including antenatal depression. The critical value was 9.5.

### Statistical Methods

Different usages of acAPPs among pregnant women were analyzed using the chi-square test. The association between acAPP usage and antenatal depression was analyzed using binary logistic regression.

## Results

### Demographic Information of Pregnant Women

The demographic characteristics of the sample and univariate analysis are shown in Table 1. The average age of the pregnant women was 28.66 (SD 3.964) years. They mainly lived in a city, were nonmigrants, had a family monthly income of 5000-10,000 yuan (US $785-1570), and had completed undergraduate or college education. More than half of the pregnant women were pregnant for the first time and about two-thirds had no previous children.

### Use of Antenatal Care Apps

In this study, 71.31% (930/1304) of the pregnant women used acAPPs. Higher usage of acAPPs was associated with urban residency, nonmigrant status, first pregnancy, planned pregnancy, having no previous children, and opportunity to communicate with peer pregnant women. There were differences in the utilization rate of acAPPs among pregnant women of different ages, family incomes, and education levels (*P*=.02, *P*=.001, *P*<.001, respectively). Usage of acAPPs among pregnant women aged 25-29 years was higher than that among women aged ≥35 years. App usage among those with family incomes in the 5000-10,000 renminbi (RMB)/month and 10,000-15,000 RMB/month brackets was higher than that among those with family incomes less than or equal to 5000 RMB/month. Usage among women with education levels of junior high school and below was lower than that among women with higher education levels (*P*<.001; see Table 2).

When choosing the kind of acAPP, pregnant women paid the most attention to the user rating and information content of acAPPs, and app stores and official websites were the main channels of acAPP downloads (Table 3). Apps commonly used by pregnant women are shown in Table 4. Baobaoshu, accounting for 45.8% of the total usage, was the most commonly used acAPP among pregnant women, followed by Meiyou and Huaiyunguanjia (14.9% and 7.4%, respectively). Other apps included Haoyunma, Yunqiguanjia, Yunqitixing, Qinbaobao, Lamabang, and more than 10 other kinds of acAPPs.

**Figure 2:**
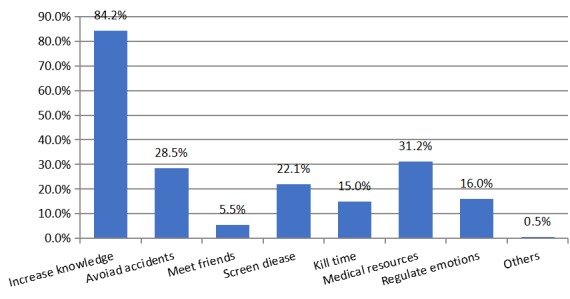
Benefits of using antenatal care apps.

Among the 10 possible functions of the health care acAPP during pregnancy, antenatal care tips, health information, changes in pregnancy records, and dietary recommendations were the most commonly used functions by pregnant women (Table 5). Moreover, 67.1% (619/930) of the pregnant women started using the acAPP mainly 12 weeks after pregnancy. Almost half (455/930, 49.1%) used acAPPs 1-2 times per day, the average duration of each use was mainly 15 minutes, and 70.86% (659/930) of the pregnant women had been using acAPPs for 3-12 months (Table 6). Comprehensive, informative, user-friendly, and reliable content were features most users said appealed to them about acAPPs. Increasing knowledge was the greatest benefit pregnant women reported receiving from using acAPPs (Figure 2).

### Antenatal Depression and Its Association With Using Antenatal Care Apps

Among pregnant women, 46.16% (602/1304) screened positive for depression by EPDS. Controlling for demographic characteristics and pregnancy situations, logistic regression showed that functions of and time spent on these apps were associated with the incidence of antenatal depression (Table 7). Moreover, acAPPs containing disease-screening functions and using apps for more than 30 minutes at a time were positively related to the occurrence of depression. AcAPPs containing social functions were negatively correlated with depression.

**Table 1:** Demographic information of pregnant women.

Item		Value, n (%)
**Age, years**		
	<25	146 (11.1)
	25-29	685 (52.1)
	30-34	322 (24.5)
	≥35	126 (9.6)
	Missing	35 (2.7)
**Residence**		
	City	858 (65.3)
	Rural area	310 (23.6)
	Missing	146 (11.1)
**Migrant population**		
	Yes	313 (23.8)
	No	933 (71.0)
	Missing	68 (5.2)
**Family income, renminbi/month (US $/month)**		
	≤5000 yuan (785)	252 (19.2)
	5000-10,000 yuan (785-1570)	674 (51.3)
	10,000-15,000 yuan (1570-2355)	192 (14.6)
	>15,000 yuan (2355)	110 (8.4)
	Missing	86 (6.5)
**Education level**		
	Junior high school and below	80 (6.1)
	High school or secondary school	236 (18.0)
	Undergraduate or college	729 (60.3)
	Master’s and above	101 (7.7)
	Missing	105 (8.0)
**First pregnancy**		
	Yes	713 (56.4)
	No	552 (42.0)
	Missing	49 (3.7)
**Planned pregnancy**		
	Yes	841 (64.0)
	No	431 (32.8)
	Missing	42 (3.2)
**Previous children**		
	Yes	427 (32.5)
	No	865 (65.8)
	Missing	22 (1.7)
**Opportunity to communicate with peer pregnant women**		
	Have	1054 (80.2)
	Do not have	245 (18.6)
	Missing	15 (1)

**Table 2:** Different usages of antenatal care apps among pregnant women.

Item		Using antenatal care app, n (%)	Not using antenatal care app, n (%)	χ^2^	*df* ^a^	*P* value
**Age, years**				9.6	3	0.02
	<25	100 (11.0)	46 (12.5)			
	25-29	500 (54.8)	185 (50.4)			
	30-34	236 (25.9)	86 (23.4)			
	≥35	76 (8.3)	50 (13.6)			
**Residence**				11.7	1	0.001
	City	636 (76.3)	222 (66.5)			
	Rural area	198 (23.7)	112 (33.5)			
**Migrant population**				11.2	1	0.001
	Yes	203 (22.6)	110 (31.7)			
	No	696 (77.4)	237 (68.3)			
**Family income, renminbi/month (US $/month)**				17.3	3	0.001
	≤5000 yuan (785)	155 (17.6)	97 (28.1)			
	5000-10,000 yuan (785-1570)	506 (57.3)	168 (48.7)			
	10,000-15,000 yuan(1570-2355)	142 (16.1)	50 (14.5)			
	>15,000 yuan (2355)	80 (9.1)	30 (8.7)			
**Education level**				56.5	3	<0.001
	Junior high school and below	30 (3.5)	50 (14.2)			
	High school or secondary school	155 (18.1)	81 (23.0)			
	Undergraduate or college	590 (68.8)	202 (57.4)			
	Master’s and above	82 (9.6)	19 (5.4)			
**First pregnancy**				8.8	1	0.003
	Yes	531 (59.0)	182 (49.9)			
	No	369 (41.0)	183 (50.1)			
**Planned pregnancy**				4.9	1	0.03
	Yes	616 (68.0)	225 (61.5)			
	No	290 (32.0)	141 (38.5)			
**Previous children**				12.2	1	<0.001
	Yes	278 (30.2)	149 (40.3)			
	No	664 (69.8)	221 (59.7)			
**Opportunity to communicate with peer pregnant women**				10.9	1	0.001
	Have	774 (83.4)	280 (75.5)			
	Did not have	154 (16.6)	91 (24.5)			

^a^*df*: degrees of freedom.

**Table 3:** Download channels of antenatal care apps.

Download variables		Value, n (%)
**Emphasis when selecting antenatal care app**		
	Information content	490 (53.1)
	User rating	385 (41.7)
	Comprehensive ranking	178 (19.3)
	Download times	137 (14.8)
	Scoring	116 (12.6)
	Other	19 (2.1)
	Memory occupied	14 (1.5)
**Download channel**		
	App stores	548 (59.7)
	Official website search	333 (36.3)
	Pop-up connection	19 (2.1)
	Other	18 (2.0)

**Table 4:** Common antenatal care apps.

App name	Value, n (%)
Baobaoshu	426 (45.8)
Meiyou	139 (14.9)
Huaiyunguanjia	69 (7.4)
Mamabang	42 (4.5)
Yunqibanlv	21 (2.2)
Baobaozhidao	21 (2.2)
Yunqitixing	15 (1.6)
Others	45 (4.8)

**Table 5:** Common functions of antenatal care apps.

Functions	Common functions, n (%)
Health knowledge education	134 (33.3)
Changes during pregnancy record	104 (25.9)
Antenatal care tips	94 (23.5)
Diet recommendations	88 (21.9)
Answers and questions	37 (9.2)
Instant communication	21 (5.2)
Trading platform	17 (4.2)
Disease screening	11 (2.7)
Hospital related	9 (2.2)
Make friends	5 (1.2)

**Table 6:** Timing of using antenatal care apps.

Time		Value, n (%)
**Start time**		
	Before pregnancy	189 (20.5)
	Within 12 weeks of gestation	619 (67.1)
	12-27 weeks of gestation	103 (11.2)
	After 27 weeks of pregnancy	12 (1.3)
**Frequency**		
	1-7 times/week	326 (35.2)
	1-2 times/day	457 (49.4)
	≥3 times/day	128 (13.8)
	Other	15 (1.6)
**Average duration of each use**		
	≤15 minute	455 (49.1)
	15-30 minute	394 (42.5)
	30-60 minute	56 (6.0)
	≥1 hour	21 (2.3)
**Longest duration of antenatal care app retainment**		
	<3 months	188 (20.4)
	3-6 months	350 (38.0)
	6-12 months	309 (33.5)
	>1 year	75 (8)

**Table 7:** Factors influencing antenatal depression.

Factors		B	Wals	*df* ^a^	*P* value	Odds ratio	95% CI	
							Lower	Upper
**Disease-screening function**								
	No	—	—	1	—	—	1.03	3.06
	Yes	0.57	4.26	1	0.04	1.78		
**Social function**								
	No	—	—	1	—	—	0.12	0.89
	Yes	−1.10	4.76	1	0.03	0.33		
**Average duration of each use**								
	<30 min	—	—	1	—	—	1.19	3.52
	≥30 min	0.72	6.78	1	0.009	2.05		
**Education level**								
	High school or secondary school	−0.96	4.28	1	0.04	0.04	0.15	0.95
	Undergraduate or college	−1.04	5.61	1	0.02	0.35	0.15	0.84
	Master’s and above	−1.32	7.25	1	0.007	0.27	0.10	0.70
	Constant	1.82	2.38	1	0.12	6.19	—	—

^a^*df*: degrees of freedom.

## Discussion

This study was a cross-sectional survey carried out in Changsha, China, which revealed that the usage of acAPPs was very high (930/1304, 71.40% of the participants had used one) and frequent (457/930, 49.4% of the users used it 1-2 times per day) among pregnant women in urban areas. After controlling for several confounding factors, functions and duration of the use of acAPPs during pregnancy were found to be related to the incidence of antenatal depression. This reminds us that when considering pregnant women’s health education, it is important to look beyond traditional pregnancy schools and maternal and child special education and recognize the usefulness of acAPPs as mobile medicine is expected to become a new means of health education and management during pregnancy [9,31,32]. At the same time, acAPP design can be optimized by adding functions, such as disease screening, peer communication, and time reminders, to increase population coverage for pregnant women and mental health care.

This study revealed 4 important aspects of the use of acAPPs. First, it confirmed that mobile apps are becoming major ways to obtain health information [33]. Research in Australia [12] involving 410 women has found that nearly three-quarters of women used pregnancy-related apps and that most of them used these apps at least once a week. A Chinese study [6] found that 78.81% of expectant mothers downloaded pregnancy-related apps. These results are similar to our findings in terms of acAPP usage rate. Compared with traditional methods of information acquisition, antenatal care apps make it possible for pregnant women to find pregnancy care knowledge at their own time, free from the restrictions of work, life, transportation, and family environment, via broad availability of smartphones and network coverage. This could help save health service costs [34,35].

Second, just as the literature [12] has shown, pregnant women with different characteristics use acAPPs differently. In this study, family income, place of residence, education, first pregnancy, planned pregnancy, having no previous children, and opportunity to communicate with pregnant women of the same age affected the usage of acAPPs. These findings suggest that we should tailor the design of acAPPs to meet the characteristics of pregnant women. Furthermore, as vulnerable groups (older, lower income and education, and unplanned pregnancies) in general tend to use acAPPs less, acAPPs would need to be better designed to reach these populations.

Third, the process of selecting an acAPP is different from routine health care decisions that are more influenced by health care professionals. Most pregnant women rely on user evaluations to select acAPPs rather than professional recommendations, which makes it particularly important for acAPPs to be professional and accurate in terms of information content. In contrast to other entertainment apps, user evaluations of acAPPs are often comments on user-friendliness, and they do not discuss professionalism, accuracy, or comprehensiveness. Studies have shown that if the pregnancy information shared by apps is inaccurate and unreliable, users of these apps face risks in terms of pregnancy protection [36]. The regulation of health acAPPs is an important issue raised in the existing research [37,38]. There are also studies [39,40] suggesting that the incorporation of a medical editorial team can increase the reliability of the knowledge provided in acAPPs in order to meet the requirements of information demanders.

Finally, acAPPs may be designed to monitor and intervene in perinatal depression [41]. This study found that scores for depression were higher in pregnant women who focused on using the acAPP’s disease-screening functions. This may be because this group of pregnant women had underlying diseases or anxieties about their own health, leading their depressive symptoms to be more pronounced. The pregnant women who preferred acApps with social functions had a desire for communication, and the use of acAPPs also increased the opportunities for communication with other pregnant women and channels of release. Pregnant women can receive mutual support and comfort [42], rendering them relatively less at risk of depressive symptoms. Studies have confirmed that peer communication is a protective factor for depressive symptoms [43]. Interestingly, the study also found that depressive symptoms were more pronounced in pregnant women who used acApps for more than half an hour. This may be because such pregnant women are isolated from the real world or have obstacles in their interpersonal relationships, leading to their immersion in the Internet [44]. This is consistent with the findings of Mansourian et al [45]. Augner et al [46,47] also found that overuse of smartphones is one of the predictors of adolescent depression. This suggests that when developers design acAPPs, proper tools, such as adding a time-use reminder function, may help users arrange rest time. AcAPPs with disease-screening functions that provide health consultations and psychological guidance could reduce the risk of antenatal depression. Adding related content and functions to acAPPs would also make them better tools for pregnancy management.

This study is a cross-sectional survey of all expecting mothers present at the Provincial Maternal and Child Care Hospital during a selected period of time; thus, data extrapolation was limited and no causal inference could be made. In addition, the self-made questionnaires used in this study were only perfected in the pre-experiment and had not been tested for reliability and validity. There is also heterogeneity in the types of antenatal care apps. In future studies, authors should consider quality evaluation and fine classification before conducting in-depth analyses. Our team used the mystery customer approach to evaluate the acAPPs’ accuracy and reliability, the results of which will be described in another article.

## Abbreviations

acAPPs: antenatal care apps
EPDS: Edinburgh Postnatal Depression Scale
